# What would other emergency stroke teams do? Using explainable machine learning to understand variation in thrombolysis practice

**DOI:** 10.1177/23969873231189040

**Published:** 2023-07-22

**Authors:** Kerry Pearn, Michael Allen, Anna Laws, Thomas Monks, Richard Everson, Martin James

**Affiliations:** 1 University of Exeter Medical School, Exeter, UK; 2 NIHR South West Peninsula Applied Research Collaboration (ARC), Plymouth, UK; 3 Computer Science, University of Exeter, Exeter, UK; 4 Royal Devon University Healthcare NHS Foundation Trust, Exeter, UK

**Keywords:** Stroke, thrombolysis, machine learning

## Abstract

**Introduction:**

The aim of this work was to understand between-hospital variation in thrombolysis use among emergency stroke admissions in England and Wales.

**Patients:**

A total of 88,928 patients who arrived at all 132 emergency stroke hospitals in England Wales within 4 h of stroke onset, from 2016 to 2018.

**Methods:**

Machine learning was applied to the Sentinel Stroke National Audit Programme (SSNAP) data set, to learn which patients in each hospital would likely receive thrombolysis. We used XGBoost machine learning models, coupled with a SHAP model for explainability; Shapley (SHAP) values, providing estimates of how patient features, and hospital identity, influence the odds of receiving thrombolysis.

**Results:**

Thrombolysis use in patients arriving within 4 h of known or estimated stroke onset ranged 7% -49% between hospitals. The odds of receiving thrombolysis reduced 9-fold over the first 120 min of arrival-to-scan time, varied 30-fold with stroke severity, reduced 3-fold with estimated rather than precise stroke onset time, fell 6-fold with increasing pre-stroke disability, fell 4-fold with onset during sleep, fell 5-fold with use of anticoagulants, fell 2-fold between 80 and 110 years of age, reduced 3-fold between 120 and 240 min of onset-to-arrival time and varied 13-fold between hospitals. The majority of between-hospital variance was explained by the hospital, rather than the differences in local patient populations.

**Conclusions:**

Using explainable machine learning, we identified that the majority of the between-hospital variation in thrombolysis use in England and Wales may be explained by differences in in-hospital processes and differences in attitudes to judging suitability for thrombolysis.

## Introduction

Stroke remains one of the leading global causes of death and disability.^1^ Despite reductions in age-standardised rates of stroke, ageing populations are driving an increase in the absolute number of strokes.^1^ Across Europe, in 2017, stroke was found to cost healthcare systems 27 billion Euros or 1.7% of health expenditure.^2^ Thrombolysis with recombinant tissue plasminogen activator can significantly reduce disability after ischaemic stroke, so long as it is given in the first few hours after stroke onset.^3^ Despite thrombolysis being of proven benefit in ischaemic stroke, use of thrombolysis varies significantly both between and within European countries.^4^ In England and Wales the national stroke audit reported that in 2021/22 thrombolysis rates for emergency stroke admissions varied from just 1% to 28% between hospitals,^5^ with a median rate of 10.4%, against a 2019 NHS England long term plan that 20% of patients of emergency stroke admissions should be receiving thrombolysis.^6^

Studies have shown that reasons for low and varying thrombolysis rates are multi-factorial. Reasons include late presentation,^4^ lack of expertise,^4^ lack of clear protocols or training,^7^ delayed access to specialists,^8^ and poor triage by ambulance or emergency department staff.^7^ For many factors, the establishment of primary stroke centres has been suggested to improve the emergency care of patients with stroke and reduce barriers to thrombolysis,^7^ with a centralised model of primary stroke centres leading to increased likelihood of thrombolysis.^9–11^

In addition to organisational factors, clinicians can have varying attitudes to which patients are suitable candidates for thrombolysis. In a discrete choice experiment,^12^ the authors concluded that there was considerable heterogeneity among respondents in their thrombolysis decision-making. Areas of difference were around whether to give thrombolysis to mild strokes, to older patients beyond 3 h from stroke onset, and when there was pre-existing disability.

Based on national audit data from 3 years of emergency stroke admissions, we have previously built models of the emergency stroke pathway,^13,14^ including using machine learning to compare thrombolysis decisions between hospitals. Using these models we found that it would be credible to target an increase in average thrombolysis in England and Wales, from 11% to 18%, but that each hospital should have its own target, reflecting differences in local populations. We found that the largest increase in thrombolysis use would come from replicating thrombolysis decision-making practice from higher to lower thrombolysing hospitals.

In our previous work we established that we could predict the use of thrombolysis in patients arriving within 4 h of known stroke onset with 84.3% accuracy.^14^ We could then ask the question ‘What if this patient attended another hospital - would they likely be given thrombolysis?’ As this was a *‘black-box’* decision-forest model we could not effectively explain the relationship between patient level data (‘features’) and their chance of receiving thrombolysis, or identify and explain the features which different hospitals would differ on.

In this paper, therefore, we seek to use *explainable machine learning* to understand the relationship between patient and hospital features and the use of thrombolysis across England and Wales, and we seek to understand how hospitals differ in their attitudes to use of thrombolysis, and how much difference in use of thrombolysis may be explained by those differences. We use an *eXtreme Gradient Boosting* (XGBoost) model^15^ to make predictions and then use an additional *SHapley Additive exPlanations*^16^ (SHAP) model to explain the contribution of each feature to the model prediction.

## Methods

Note: further details of methods may be found in the Supplemental Appendix.

### Data

Data were retrieved for 246,676 emergency stroke admissions to acute stroke teams in England and Wales for the years 2016–2018, obtained from the Sentinel Stroke National Audit Programme (SSNAP). Data fields were provided for the hyper-acute phase of the stroke pathway, up to and including our target feature: *receive thrombolysis*. Of these patients, 88,928 arrived within 4 h of known stroke onset, and were used in this modelling study. A 4 h onset-to-arrival cut-off was used to allow for 30 min for scan and thrombolysis to be within the allowed 4.5 onset-to-thrombolysis time. The data included 132 acute stroke hospitals.

### Machine learning models (to predict thrombolysis use)

We used XGBoost^15^ to predict the probability of use of thrombolysis for each patient from their other feature values.

#### Machine learning models

For the different analysis included in this paper, we trained three separate XGBoost models. Each analysis will refer to the model used:

1. *K-fold* model: A 5-fold train-test cross validation used to test the accuracy of the model, for feature selection, and to test reproducibility of SHAP values.
2. *All data* model: A single model trained on all patients, used to investigate the relationship between feature values and predictions.
3. *10k holdout* model: A model trained on all data apart from a 10k hold-out set. This model is used to mimic a 10k cohort of patients that attends all hospitals (by changing the hospital encoding) to further investigate variation in thrombolysis decision-making between hospitals.

#### Feature selection

The full dataset contains 83 features. In order to simplify the model (for enhanced explainability) we selected a subset of these features to be included in the machine learning model – those that are the most predictive of thrombolysis use. Features were selected by forward-feature selection (using the *k-fold* model), identifying one feature at a time that led to the greatest improvement in accuracy as measured by Receiver Operating Characteristic (ROC) Area Under Curve (AUC). We repeated this process, identifying the next most important feature, until the model accuracy was equivalent to the model with all 83 features. These results were used to identify the number of features to include in our machine learning models.

#### Model accuracy

Model accuracy, ROC AUC, sensitivity and specificity were measured using the *k-fold* model. Predicted thrombolysis rate was compared with actual thrombolysis use at hospital-level.

### SHapley Additive exPlanation (SHAP) values

We sought to make our models explainable using SHAP values (calculated using the SHAP library^16^). SHAP provides a measure of the contribution of each feature value to the final predicted probability of receiving thrombolysis for that individual. The SHAP values for each feature are comprised of the feature’s main effect (the effect of that feature in isolation) and all of the pairwise interaction effects with each of the other features. SHAP values provide the influence of each feature as the change in log-odds of receiving thrombolysis (SHAP values expressed as log-odds are additive). SHAP was calculated for each of our three XGBoost models: *k-fold, all data* and *10k holdout* model.

### The relationship between feature values and the odds of receiving thrombolysis

For each feature, we examined the relationship between feature values and their corresponding SHAP values (we used values from the *all data* model).

### Investigating how the identity of a hospital influences thrombolysis rate

For each hospital we compared the mean SHAP main effect value for the hospital attended (using values from the *all data* model) with the hospitals observed thrombolysis use.

To reveal the variation in thrombolysis rate due to hospital, rather than patient mix, we also compared the mean hospital attended SHAP main effect value for the identical 10k patient cohort attending each hospital, with the hospitals’ predicted thrombolysis use for this 10k patient cohort (we used values from the *10k holdout* model).

### Investigating how patient populations and hospital identity and processes influences thrombolysis rate

The 10 features in the model can be classified into two subsets: (1) ‘patient descriptive features’ (features that describe the patients characteristics) and (2) ‘hospital descriptive features’ (features that describe the hospital’s identity or processes). To analyse the influence that each subset of features has on the thrombolysis rate, using values from the *all data* model, we calculated the ‘subset SHAP value’ for each feature, which only includes the components of its SHAP value that contain effects from the features in the same subset. This is expressed as the sum of the main effect and the interaction effects with the other features in the same subset. Multiple regression models were then fitted to the mean subset SHAP values.

### Variation in hospital thrombolysis use for patient subgroups

Informed by the SHAP values, we analysed the observed and predicted use of thrombolysis in 11 subgroups of patients: one subgroup for ‘ideally’ thrombolysable patients, nine ‘sub-optimal’ thrombolysable patient subgroups (one subgroup per feature), and one subgroup with two sub-optimal features. The 11 patient subgroups were defined as:

1. An *‘ideally’* thrombolysable patient:
  ● Stroke caused by infarction
  ● Arrival-to-scan time <30 min
  ● NIHSS in range 10–25
  ● Precise stroke onset time known
  ● No pre-stroke disability (modified Rankin Scale, mRS, 0)
  ● Not taking atrial fibrillation anticoagulants
  ● Onset-to-arrival time <90 min
  ● Age<80 years old
  ● Onset not during sleep
2. Haemorrhagic stroke
3. Arrival-to-scan time 60–90 min
4. NIHSS <5
5. Estimated stroke onset time
6. Existing pre-stroke disability (mRS > 2)
7. Using atrial fibrillation anticoagulants
8. Onset-to-arrival time 150–180 min
9. Age 80+ years old
10. Onset during sleep
11. NIHSS <5 *and* with estimated stroke onset time

The observed thrombolysis use for each subgroup at each hospital was taken from the SSNAP dataset. In order to further reveal the variation in thrombolysis use that was due to hospital decision-making we predicted thrombolysis use for the same patient subgroups at each hospital by using the *10k holdout* model.

## Results

### Variation in observed hospital thrombolysis use

Thrombolysis use in the original data varied between hospitals from 1.5% to 24.3% of all patients, and 7.3% to 49.7% of patients arriving within 4 h of known (precise or estimated) stroke onset.

### Feature selection

A model using 10 features had an ROC AUC of 0.923, compared to 0.922 for a model using all of the 83 available features. We selected 10 features for all subsequent work, which were (in order of selection):

- *Arrival-to-scan time* (min)
- *Infarction*: Stroke type (1 = infarction, 0 = haemorrhage)
- *Stroke severity* (NIHSS on arrival)
- *Precise onset time* (1 = precise, 0 = best estimate)
- *Prior disability level* (mRS before stroke)
- *Stroke team*
- *Use of anticoagulants* (prior to stroke onset; 1 = Yes, 0 = No)
- *Onset-to-arrival time* (min)
- *Onset during sleep* (1 = Yes, 0 = No)
- *Age* (midpoint of 5 year age bands)

Correlations between the 10 features were measured. All *r*-squared were less than 0.05 except (a) age and prior disability level (*r*-squared 0.146) and (b) onset during sleep and precise onset time (*r*-squared 0.078).

#### Model accuracy

Model accuracy was measured using stratified 5-fold cross validation. Overall accuracy was 85.0% (83.9% sensitivity and specificity could be achieved simultaneously). The model predicted hospital thrombolysis use at each hospital with very good accuracy (*r*-squared = 0.977, with a mean absolute error of 1.1 percentage points). The Appendix contains further model accuracy analysis.

### Individual patient SHAP values

**Figure 1.**
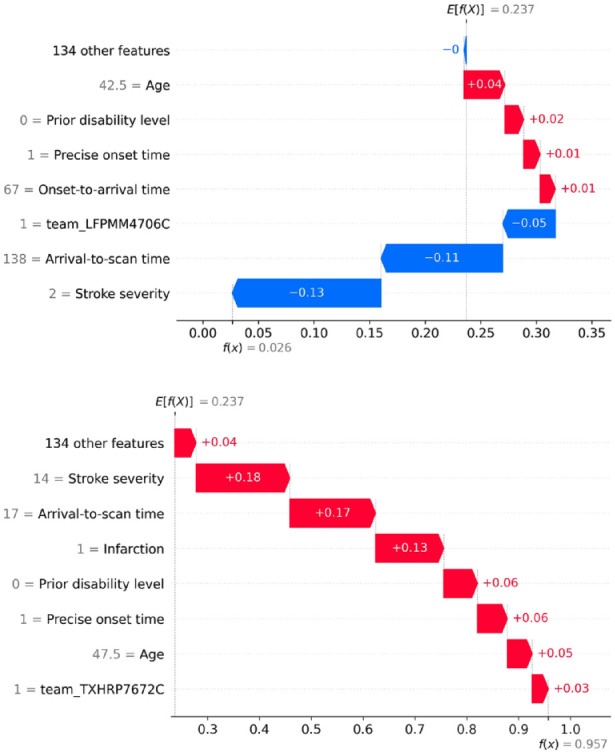
Waterfall plots showing the influence of each feature on the predicted probability of a single patient receiving thrombolysis. Top: An example of a patient with a low probability (2.6%) of receiving thrombolysis. Bottom: An example of a patient with a high probability (95.7%) of receiving thrombolysis.

SHAP values are calculated as how they affect log odds of receiving thrombolysis, but for individual predictions, probability values are more intuitive. Figure 1 shows *waterfall* plots for example patients with low and high probability of receiving thrombolysis. Waterfall plots show the influence of features for an individual prediction (in our case, patient). The SHAP model starts with a base prediction of a 24% probability of receiving thrombolysis, before feature values are taken into account. For the patient with a low probability of receiving thrombolysis, the two most influential features reducing the probability of thrombolysis were a long arrival-to-scan time (138 min) and a low stroke severity (NIHSS = 2). For the patient with a high probability of receiving thrombolysis, the two most influential features increasing the probability of thrombolysis were a short arrival-to-scan time (17 min) and a moderate stroke severity (NIHSS = 14).

### The relationship between feature values and the odds of receiving thrombolysis

**Figure 2.**
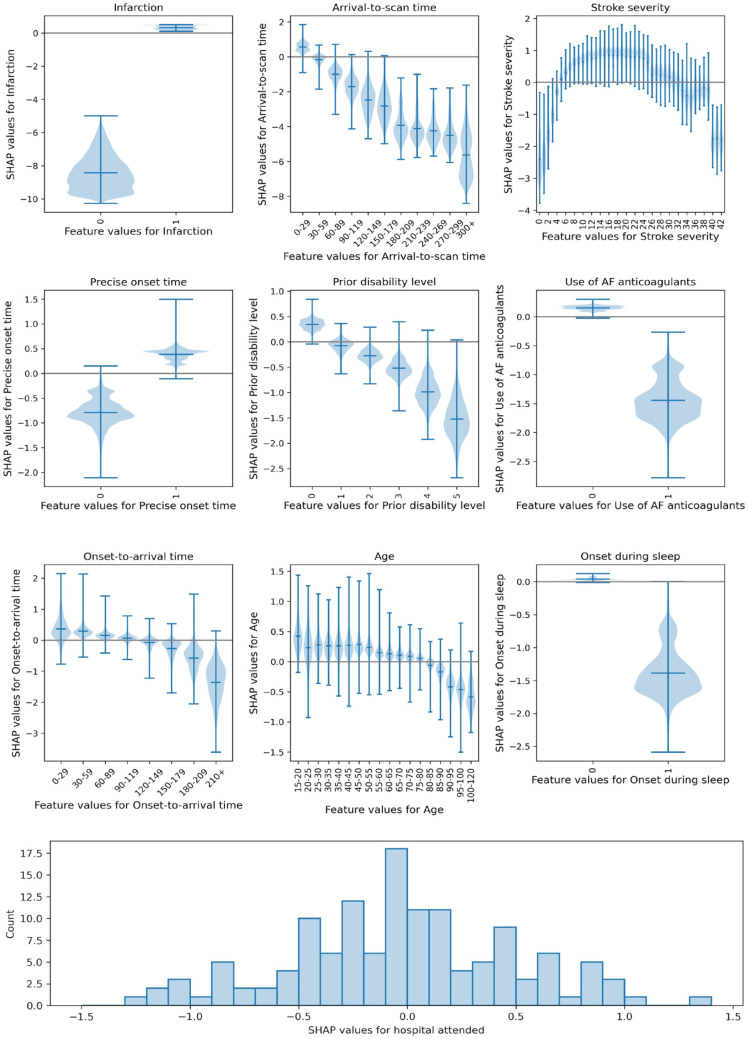
Plots showing the relationship between SHAP values and feature values. Top: Violin plots showing the relationship between SHAP values and feature values. The horizontal line shows the median SHAP value. The plots are ordered in ranked feature importance (using the mean absolute SHAP value across all instances). Bottom: Histogram showing the frequency of SHAP values for the hospital attended.

Figure 2 shows the relationship between patient level feature values and their SHAP values. Key observations are:

- *Stroke type*: The SHAP values for stroke type show that the model effectively eliminated any probability of receiving thrombolysis for haemorrhagic stroke.
- *Arrival-to-scan time*: The odds of receiving thrombolysis reduced by 9-fold over the first 120 min of arrival-to-scan time.
- *Stroke severity (NIHSS)*: The odds of receiving thrombolysis were lowest at NIHSS 0, increased and peaked at NIHSS 15–25, and then fell again with higher stroke severity (NIHSS above 25). The difference between minimum odds and maximum odds of receiving thrombolysis was 30-fold.
- *Stroke onset time type*: The odds of receiving thrombolysis were 3-fold greater for precise onset time than estimated onset time.
- *Disability level (mRS) before stroke*: The odds of receiving thrombolysis fell 6-fold between mRS 0 and 5.
- *Use of AF anticoagulants*: The odds of receiving thrombolysis were reduced 5-fold with anticoagulant use.
- *Onset-to-arrival time*: The odds of receiving thrombolysis were similar below 120 min, then fell 3-fold between 120 and 240 min.
- *Age*: The odds of receiving thrombolysis were similar below 80 years old, then fell 2-fold between 80 and 110 years old.
- *Onset during sleep*: The odds of receiving thrombolysis were 4-fold lower for onset during sleep.
- *Hospital attended*: There was a 13-fold difference in odds of receiving thrombolysis between hospitals.

### Investigating how the identity of a hospital influences thrombolysis rate

**Figure 3.**
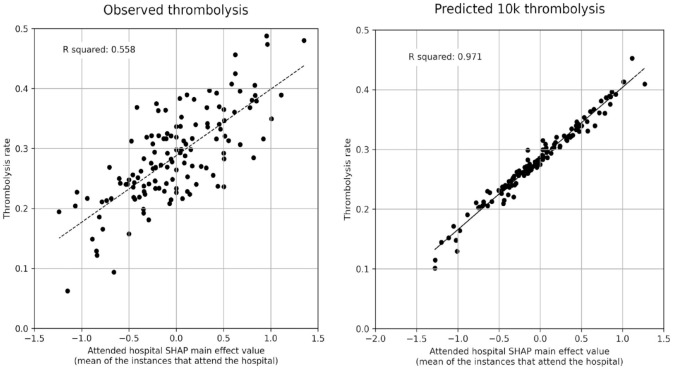
Correlations between hospital SHAP main effect value and the observed thrombolysis use at each hospital. Left: Observed thrombolysis (using the all data model). Right: Predicted 10k cohort thrombolysis rate (using the 10k holdout model).

The mean hospital SHAP main effect value correlated with the observed hospital thrombolysis rate with an r-squared of 0.558 (Figure 3, left), suggesting that 56% (*p* = 0.0001) of the between-hospital variance in thrombolysis use may be explained by the attended hospitals’ SHAP main effect values, that is, the hospitals’ predisposition and/or preparedness to use thrombolysis.

Using the *10k holdout* model, the predicted use of thrombolysis across the 132 hospitals for the identical 10k cohort of patients ranged from 10% to 45%. The mean hospital SHAP main effect value for the 10k cohort correlated very closely with the predicted thrombolysis use in the 10k cohort at each hospital (r-squared of 0.971, Figure 3, right), confirming that the hospital SHAP main effect value is providing direct insight into hospitals’ propensity to use thrombolysis.

We performed further analysis on the relationship of hospital admission numbers and hospital process speeds with hospital SHAP values. The strongest relationship found was that median scan-to-thrombolysis time was correlated with mean hospital SHAP value with r-squared of 0.156 (*p* < 0.01), with shorter scan-to-arrival times having a higher hospital SHAP value. Admission numbers was weakly correlated with mean hospital SHAP value (*r*-squared = 0.047, *p* = 0.012), with higher admission numbers having a higher hospital SHAP value. Median arrival-to-scan time was not correlated with mean hospital SHAP value (*p* > 0.05). See appendix for more details.

### Investigating how patient populations and hospital identity and processes influences thrombolysis rate

**Figure 4.**
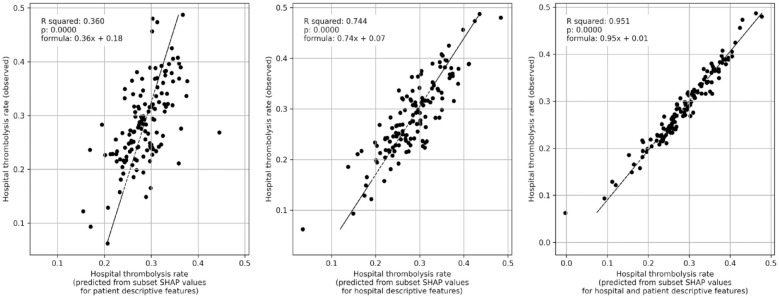
Multiple regression of subset SHAP values (mean of patients attending hospital) with hospital observed thrombolysis rate (using the all data model). Left: Subset SHAP values for the eight patient descriptive features (age, stroke severity, prior disability, onset-to-arrival time, stroke type, type of onset time, anticoagulants, and onset during sleep). Middle: Subset SHAP values for the two hospital descriptive features (arrival-to-scan time, and hospital attended). Right: Subset SHAP values for all 10 features (for both hospital and patient descriptive features).

We predicted thrombolysis use using mean subset SHAP values for patients attending each hospital. Figure 4 shows that 36% (*p* = 0.0001) of the variance in observed between-hospital thrombolysis use can be explained by the patient population, 74% (*p* = 0.0001) can be explained by hospital identity and processes, and that 95% (*p* = 0.0001) can be explained by the combined information from both the patient population and hospital identity and processes.

### Variation in hospital thrombolysis use for patient subgroups

**Figure 5.**
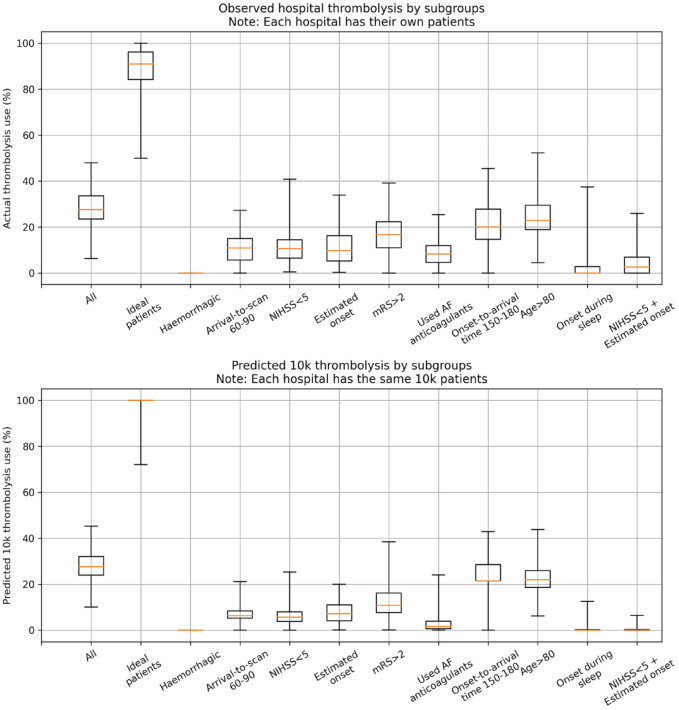
Boxplot for either observed (top) or predicted (bottom) use of thrombolysis for subgroups of patients. The ‘ideal patients’ subgroup has a mid-level stroke severity (NIHSS 10–25), short arrival-to-scan time (<30 min), stroke caused by infarction, precise stroke onset time known, no pre-stroke disability (mRS 0), not taking any atrial fibrillation anticoagulants, short onset-to-arrival time (<90 min), <80 years old, and onset not during sleep. The single features are ordered in ranked feature importance (using the mean absolute SHAP value across all instances).

Figure 5 shows observed and predicted use of thrombolysis, broken down by patient subgroup. The subgroups of patients with one defined non-ideal feature all had reduced thrombolysis use than the complete patient population, and combining these non-ideal features reduced thrombolysis use further. There was, however, significant variation between hospitals’ thrombolysis use in each of these subgroups. The observed and predicted thrombolysis use show the same general patterns.

## Discussion

We have built on our previous work to predict thrombolysis use from patient level data, by creating an *explainable machine learning model* which maintains the high accuracy that we previously achieved (85%).^14^ Predicted thrombolysis use at each hospital also very closely matched observed thrombolysis use. The SSNAP registry data used therefore appears to contain most of the information used to make thrombolysis decisions in clinical practice, and can explain the very large majority of between-hospital variation in thrombolysis use.

In general, using SHAP values to uncover the relationship between patient characteristics and the probability of receiving thrombolysis, we found that the probability of receiving thrombolysis fell with increasing arrival-to-scan times, was dependent on stroke severity with the probability of receiving thrombolysis being highest between NIHSS 10 and 25, was lower when onset time was estimated rather than known precisely, and fell with increasing disability prior to stroke. These patterns are similar to the observations of a discrete choice experiment with hypothetical patients,^12^ but in our study we confirm these patterns in actual use of thrombolysis, can be quantitative about the effect, and we add the importance of time-to-scan and whether an onset time is known precisely.

Hospital SHAP values correlated very closely with the predicted use of thrombolysis in a 10k cohort of patients, confirming that the hospital SHAP main effect value provides a measure of the predisposition of a hospital to use thrombolysis. We found that hospital identity and processes explained 74% of the variance in observed thrombolysis for patients arriving in time to receive thrombolysis. There was a slight tendency for larger hospitals to have a higher hospital SHAP value, and a stronger tendency for hospitals with shorter scan-to-thrombolysis times to have a higher hospital SHAP value. This suggests that those hospitals with a higher propensity to give thrombolysis are associated with a higher preparedness to give thrombolysis.

After observing the general patterns that exist in the use of thrombolysis, we created a subgroup of patients reflecting what appeared to be an *ideal* candidate for thrombolysis, and also a subgroup per feature where we expected to see lower use of thrombolysis. Observed thrombolysis in these groups reflected the patterns identified by the SHAP analysis. For the *ideal* candidates of thrombolysis, half of stroke units would give thrombolysis to at least 90% of these patients, but some units gave it to significantly fewer patients. Use of thrombolysis in the other subgroups of patients was, as expected, lower, but use also varied significantly between hospitals. Hospitals have different levels of tolerance for non-ideal patient characteristics. These patterns, of lower but varying use, were repeated with expected use of thrombolysis in the same 10k patient cohort of patients.

This novel analysis examines and aids understanding of between-hospital variation in clinical decision making in the acute stroke setting. The use of large datasets such as SSNAP to understand sources of variation in clinical practice between large number of acute stroke centres across the UK presents a unique opportunity to understand the specific influences behind the significant residual between-hospital variation in thrombolysis use. In particular, it allows national quality improvement projects such as SSNAP to counter one of the most common objections raised to comparative audit: that the patients presenting to any one particular site are in some way unique, thereby accounting for most of the variation in clinical quality between that site and all the others. Although the patient population does vary between hospitals, and will contribute to the thrombolysis use achievable by an individual hospital, the majority of between-hospital variation can be explained by hospital-level rather than patient-level factors.

It is disappointing that even though this disability-saving treatment was first licenced for approval for use over 20 years ago, it is still subject to such large variation in clinical judgement or opinion regarding the selection of patients most appropriate for use. In our previous work,^13^ we have shown that increasing the uptake of thrombolysis through the administration of treatment to more patients and sooner after stroke, offers the prospect of more than doubling the proportion of patients after stroke who are left with little or no disability (mRS 0 or 1). At a time when there is an appropriate focus of effort on expanding the use of endovascular therapy in acute ischaemic stroke, it is sobering to consider how much population benefit there still remains to accrue from the fullest possible implementation of a cheaper technology that has been available for over 20 years. Far greater scrutiny of such residual variation in clinical practice is clearly warranted, given the extent to which it appears to be acting as a barrier to successful implementation. Recent studies have highlighted that clinicians can be reluctant to modify their behaviour in response to audit and feedback when it is not seen to be clinically meaningful, recent or reliable,^17^ so the full potential of audit and feedback is not realised^18^ despite the evidence of a beneficial effect especially when baseline performance is low.^19^ The development of bespoke, individualised feedback (at least at hospital level) based on actual and recent activity may increase the impact of efforts at data-driven quality improvement targeted at increasing overall uptake of thrombolysis through reducing variation.

## Limitations

This machine learning study is necessarily limited to data collected for the national stroke audit. Though we have high accuracy, and can identify clear patterns of use of thrombolysis, the data will not be sufficient to provide a decision-support tool or to review decision-making at an individual patient level. Nor is it a causal model. We may also be missing information that could otherwise have improved the accuracy still further. The model has high accuracy and can identify clear patterns, suggesting the capability to identify and characterise a centre’s culture in the use of thrombolysis, but we do not identify variation in thrombolysis between individual clinicians in the same hospital.

We acknowledge that not all countries have a national stroke audit dataset, however we hope that this paper helps to demonstrate what type of analysis can be done should resources be allocated to collect their national data.

## Supplemental Material

sj-pdf-1-eso-10.1177_23969873231189040 – Supplemental material for What would other emergency stroke teams do? Using explainable machine learning to understand variation in thrombolysis practice Click here for additional data file. Supplemental material, sj-pdf-1-eso-10.1177_23969873231189040 for What would other emergency stroke teams do? Using explainable machine learning to understand variation in thrombolysis practice by Kerry Pearn, Michael Allen, Anna Laws, Thomas Monks, Richard Everson and Martin James in European Stroke Journal

sj-pdf-2-eso-10.1177_23969873231189040 – Supplemental material for What would other emergency stroke teams do? Using explainable machine learning to understand variation in thrombolysis practice Click here for additional data file. Supplemental material, sj-pdf-2-eso-10.1177_23969873231189040 for What would other emergency stroke teams do? Using explainable machine learning to understand variation in thrombolysis practice by Kerry Pearn, Michael Allen, Anna Laws, Thomas Monks, Richard Everson and Martin James in European Stroke Journal
