# Supplementary material for: What would other emergency stroke teams do? Using explainable machine learning to understand variation in thrombolysis practice
Source: Eur Stroke J. 2023 Jul 22;8(4):956–65. doi: 10.1177/23969873231189040 (PMC10683721; doi:10.1177/23969873231189040)
Supplement: sj-pdf-1-eso-10.1177_23969873231189040 – Supplemental material for What would other emergency stroke teams do? Using explainable machine learning to understand variation in thrombolysis practice [file sj-pdf-1-eso-10.1177_23969873231189040.pdf]

A Appendix

This work is licensed under a Creative Commons (BY:) 3.0 license.

All code, with detailed results, used is available online as a Jupyter book at `https://samuel-book.github.io/samuel_shap_paper_1/` and available on GitHub at `https://github.com/samuel-book/samuel_shap_paper`

A.1 Data

A.1.1 Data access

Data was obtained from the Sentinel Stroke National Audit (SSNAP[1]), managed through the Healthcare Quality Improvement Partnership (HQIP[2]). SSNAP has near-complete coverage of all acute stroke admissions in the UK (outside Scotland). All hospitals admitting acute stroke participate in the audit, and year-on-year comparison with Hospital Episode Statistics[3] confirms estimated case ascertainment of 95% of coded cases of acute stroke.

The NHS Health Research Authority decision tool[4] was used to confirm that ethical approval was not required to access the data. Data access was authorised by HQIP (reference HQIP303).

Data were retrieved for 246,676 emergency stroke admissions to acute stroke teams in England and Wales for the three calendar years 2016 - 2018, obtained from the Sentinel Stroke National Audit Programme[5] (SSNAP). Data fields were provided for the hyper-acute phase of the stroke pathway, up to and including our target feature: *receive thrombolysis* (full details of the data fields obtained are provided in the appendix). Of these patients, 88,928 arrived within 4 hours of known (precise or estimated) stroke onset, and were used in this modelling study. The data included 132 acute stroke hospitals (these were all units admitting an average of 100 patients per year, and delivering thrombolysis to at least 10 patients over 3 years). There are 60 original features used from the SSNAP dataset.

SSNAP has near-complete coverage of all acute stroke admissions in the UK (outside Scotland). All hospitals admitting acute stroke participate in the audit, and year-on-year comparison with Hospital Episode Statistics[6] confirms estimated case ascertainment of 95% of coded cases of acute stroke.

A.1.2 Data fields

Stroke Team

- *StrokeTeam*: Pseudonymised SSNAP 'routinely admitting team' unique identifier. For emergency care it is expected that each hospital has one stroke team (though post-72 hour care may be reported under a different team at that hospital).

Patient – general

- *Pathway*: Total number of team transfers, excluding community teams
- *S1AgeOnArrival*: Age on arrival aggregated to 5 year bands
- *MoreEqual80y*: Whether the patient is >= 80 years old at the moment of the stroke
- *S1Gender*: Gender

[1] https://www.strokeaudit.org/
[2] https://www.hqip.org.uk/
[3] https://digital.nhs.uk/data-and-information/data-tools-and-services/data-services/hospital-episode-statistics
[4] http://www.hra-decisiontools.org.uk/research/
[5] https://www.strokeaudit.org/
[6] https://digital.nhs.uk/data-and-information/data-tools-and-services/data-services/hospital-episode-statistics

- *S1Ethnicity*: Patient Ethnicity. Aggregated to White, Black, Mixed, Asian and Other

Patient – pathway information

- *S1OnsetInHospital*: Whether the patient was already an inpatient at the time of stroke
- *S1OnsetToArrival_min*: Time from symptom onset to arrival at hospital in minutes, where known and if out of hospital stroke
- *S1OnsetDateType*: Whether the date of onset given is precise, best estimate or if the stroke occurred while sleep
- *S1OnsetTimeType*: Whether the time of symptom onset given is precise, best estimate, not known
- *S1ArriveByAmbulance*: Whether the patient arrived by ambulance
- *S1AdmissionHour*: Hour of arrival, aggregates to 3 hour epochs
- *S1AdmissionDay*: Day of week at the moment of admission
- *S1AdmissionQuarter*: Year quarter (Q1: Jan-Mar; Q2:April-Jun; Q3: Jul-Sept; Q4: Oct-Dec)
- *S1AdmissionYear*: Year of admission
- *S2BrainImagingTime_min*: Time from Clock Start to brain scan. In minutes. "Clock Start" is used throughout SSNAP reporting to refer to the date and time of arrival at first hospital for newly arrived patients, or to the date and time of symptom onset if patient already in hospital at the time of their stroke.
- *S2ThrombolysisTime_min*: Time from Clock Start to thrombolysis. In minutes. "Clock Start" is used throughout SSNAP reporting to refer to the date and time of arrival at first hospital for newly arrived patients, or to the date and time of symptom onset if patient already in hospital at the time of their stroke.

Patient – comorbidities

- *CongestiveHeartFailure*: Pre-Stroke Congestive Heart Failure
- *Hypertension*: Pre-Stroke Systemic Hypertension
- *AtrialFibrillation*: Pre-Stroke Atrial Fibrillation (persistent, permanent, or paroxysmal)
- *Diabetes*: Comorbidities: Pre-Stroke Diabetes Mellitus
- *StrokeTIA*: Pre-Stroke history of stroke or Transient Ischaemic Attack (TIA)
- *AFAntiplatelet*: Only available if "Yes" to Atrial Fibrillation comorbidity. Whether the patient was on antiplatelet medication prior to admission
- *AFAnticoagulent*: Prior to 01-Dec-2017: Only available if "Yes" to Atrial Fibrillation comorbidity; From 01-Dec-2017: available even if patient is not in Atrial Fibrillation prior to admission. Whether the patient was on anticoagulant medication prior to admission
- *AFAnticoagulentVitK*: If the patient was receiving anticoagulant medication, was it vitamin K antagonists
- *AFAnticoagulentDOAC*: If the patient was receiving anticoagulant medication, was it direct oral anticoagulants (DOACs)
- *AFAnticoagulentHeparin*: If the patient was receiving anticoagulant medication, was it Heparin

Patient – NIH Stroke Scale

- *S2NihssArrival*: National Institutes of Health Stroke Scale score on arrival at hospital
- *BestGaze*: National Institutes of Health Stroke Scale Item 2 Best Gaze (higher values indicate more severe deficit)
- *BestLanguage*: National Institutes of Health Stroke Scale Item 9 Best Language (higher values indicate more severe deficit)
- *Dysarthria*: National Institutes of Health Stroke Scale Item 10 Dysarthria (higher values indicate more severe deficit)
- *ExtinctionInattention*: National Institutes of Health Stroke Scale Item 11 Extinction and Inattention (higher values indicate more severe deficit)
- *FacialPalsy*: National Institutes of Health Stroke Scale Item 4 Facial Paresis (higher values indicate more severe deficit)
- *LimbAtaxia*: National Institutes of Health Stroke Scale Item 7 Limb Ataxia (higher values indicate more severe deficit)
- *Loc*: National Institutes of Health Stroke Scale Item 1a Level of Consciousness (higher values indicate more severe deficit)
- *LocCommands*: National Institutes of Health Stroke Scale Item 1c Level of Consciousness Commands (higher values indicate more severe deficit)
- *LocQuestions*: National Institutes of Health Stroke Scale Item 1b Level of Consciousness Questions (higher values indicate more severe deficit)
- *MotorArmLeft*: National Institutes of Health Stroke Scale Item 5a Motor Arm - Left (higher values indicate more severe deficit)
- *MotorArmRight*: National Institutes of Health Stroke Scale Item 5b Motor Arm - Right (higher values indicate more severe deficit)
- *MotorLegLeft*: National Institutes of Health Stroke Scale Item 6a Motor Leg - Left (higher values indicate more severe deficit)
- *MotorLegRight*: National Institutes of Health Stroke Scale Item 6b Motor Leg - Right (higher values indicate more severe deficit)
- *Sensory*: National Institutes of Health Stroke Scale Item 8 Sensory (higher values indicate more severe deficit)
- *Visual*: National Institutes of Health Stroke Scale Item 3 Visual Fields (higher values indicate more severe deficit)

Patient – other clinical features

- *S2INR*: Patient's International Normalised ratio (INR) on arrival at hospital (available since 01-Dec-2017)
- *S2INRHigh*: INR was greater than 10 on arrival at hospital (available since 01-Dec-2017)
- *S2INRNK*: INR not checked (available since 01-Dec-2017)
- *S2NewAFDiagnosis*: Whether a new diagnosis of Atrial Fibrillation was made on admission

- *S2RankinBeforeStroke*: Patient's modified Rankin Scale score before this stroke (Higher values indicate more disability)
- *S2StrokeType*: Whether the stroke type was infarction or primary intracerebral haemorrhage
- *S2TIAInLastMonth*: Whether the patient had a Transient Ischaemic Attack during the last month. Item from the SSNAP comprehensive dataset questions (not mandatory)

Patient – thrombolysis given

- *S2Thrombolysis*: Whether the patient was given thrombolysis (clot busting medication)

Patient – reason stated for not giving thrombolysis

- *Age*: If the answer to thrombolysis given was "no but", the reason was Age
- *Comorbidity*: If the answer to thrombolysis given was "no but", the reason was Co-morbidity
- *Haemorrhagic*: If the answer to thrombolysis given was "no but", the reason was Haemorrhagic stroke
- *Improving*: If the answer to thrombolysis given was "no but", the reason was Symptoms Improving
- *Medication*: If the answer to thrombolysis given was "no but", the reason was Medication
- *OtherMedical*: If the answer to thrombolysis given was "no but", the reason was Other medical reason
- *Refusal*: If the answer to thrombolysis given was "no but", the reason was Refusal
- *TimeUnknownWakeUp*: If the answer to thrombolysis given was "no but", the reason was Symptom onset time unknown/wake-up stroke
- *TimeWindow*: If the answer to thrombolysis given was "no but", the reason was Age
- *TooMildSevere*: If the answer to thrombolysis given was "no but", the reason was Stroke too mild or too severe

A.2 Probability, odds, and Shap values (log odds shifts): A brief explanation

Many of us find it easiest to think of the chance of something occurring as a probability. For example, there might be a probability of 10% that it will rain today. That is the same as saying there will be one rainy day out of ten days for days with this given probability of rain.

In our stroke thrombolysis model, Shap values tell us how knowing something particular about a patient (such as the patient *feature*, 'Is their stroke caused by a clot or a bleed?') adjusts our prediction of whether they will receive thrombolysis or not.

This is made a little more complicated for us because Shap is usually reported as a *log odds shift*. It is useful for us to see how those relate to probabilities, and get a sense of how significant Shap values in the range of 0.5 to 5 (or -0.5 to -5) are, as that is a common range of Shap values that we will see in our models.

A.2.1 Probability

We will take the example that Shap reports that a model's base probability prediction, before consideration of features is 0.25, or a 25% probability of receiving thrombolysis; that is 1 in 4 patients with this prediction would be expected to receive thrombolysis.

A.2.2 Odds

Probability expresses the chance of something happening as the number of positive occurrences as a fraction of all occurrences (i.e. the number of patients receiving thrombolysis as a fraction of the total number of patients).

Odds express the chance of something happening as the ratio of the number of positive occurrences (i.e. receiving thrombolysis) to the number of negative occurrences (i.e. *not* receiving thrombolysis).

If we have probability prediction of 0.25 would receive thrombolysis, that would mean 1 in 4 of those patients receive thrombolysis. Expressed as odds, for every one patient that receives thrombolysis, three will not. The odds are expressed as 1:3 or 1/3. This may also be calculated as a decimal (1 divided by 3), 0.333.

Odds (O) and probability (P) may be converted with the following equations:

(1) O = P / (1 - P)

(2) P = O / (1 + O)

A.2.3 Shap values: Log odds shifts

Here we will calculate the effect of Shap values, and try and build some intuition on the size of effect Shap values of 0.5 to 5 give (we will look at positive and negative Shap values).

Shap usually outputs the effect of a particular feature in how much it shifts the odds. For reasons we will not go into here, that shift (which is the 'Shap value') is usually given in 'log odds' (the logarithm of the odds value). For the mathematically inclined, we use the natural log (*ln*).

Let's look at some Shap values (log odds) and see how much they change the odds of receiving thrombolysis.

First we'll look at the shift in odds the Shap values give. This is calculated as *shift = exp(Shap)* (table A.1).

Table A.1: The relationship between *odds* and *log odds*.

SHAP (log odds)	Shift in odds (multiply original odds)
0.5	1.65
1	2.72
2	7.39
3	20.1
4	54.6
5	148

Positive Shap values: a worked example

Now let us work through an example of starting with a known baseline *probability* (before we consider what we know about a particular patient feature), converting that to *odds*, applying a Shap *log odds shift* for that particular feature, and converting back to *probability* after we have applied the influence of that feature.

The the effects of those shifts on our baseline probability of 0.25 are shown in table A.2.

Table A.2: The effect of SHAP values between 0.5 and 5 on a base probability of 0.25

Starting P	Starting O	SHAP	Shift (multiply O)	Shifted O	Shifted P (%)
0.25 (25%)	0.333	0.5	1.65	0.550	0.3547 (35.5%)
0.25 (25%)	0.333	1	2.72	0.907	0.4754 (47.5%)
0.25 (25%)	0.333	2	7.39	2.46	0.7112 (71.1%)
0.25 (25%)	0.333	3	20.1	6.70	0.8700 (87.0%)
0.25 (25%)	0.333	4	54.6	18.2	0.9479 (94.8%)
0.25 (25%)	0.333	5	148	49.5	0.9802 (98.0%)

So, for example, a Shap value of 0.5 for one particular feature tells us that that particular feature in that patient shifts our expected probability of that patient receiving thrombolysis from 25% to 36%. A Shap value of 5 for the same feature would shift the probability of that patient receiving thrombolysis up to 98%.

Negative Shap values: a worked example

If we have a negative Shap value then odds are reduced (a Shap of -1 will lead to the odds being divided by 2.72, which is the same as multiplying by 1/2.72, which is 0.3679), as shown in table A.3.

Table A.3: The effect of SHAP values between -0.5 and -5 on a base probability of 0.25

Starting P	Starting O	Shap	Shift (multiply O)	Shifted O	Shifted P
0.25 (25%)	0.333	-0.5	0.6065	0.2022	0.1682 (16.8%)
0.25 (25%)	0.333	-1	0.3679	0.1226	0.1092 (10.9%)
0.25 (25%)	0.333	-2	0.1353	0.0451	0.0432 (4.32%)
0.25 (25%)	0.333	-3	0.0498	0.0166	0.0163 (1.63%)
0.25 (25%)	0.333	-4	0.0183	0.0061	0.0061 (0.61%)
0.25 (25%)	0.333	-5	0.0067	0.0022	0.0022 (0.22%)

So, for example, a Shap value of -0.5 for one particular feature tells us that that particular feature in that patient shifts our expected probability of that patient receiving thrombolysis from 25% to 17%. A Shap value of 5 for the same feature would shift the probability of that patient receiving thrombolysis down to 2%.

A.2.4 Observations about Shap values

We begin to get some intuition on Shap values. A Shap value of 0.5 (or -0.5) leads to a small, but still noticeable, change in probability. Shap values of 5 or -5 have effectively pushed probabilities to one extreme or the other.

A.2.5 Limitations of SHAP

SHAP is a popular method for explaining the predictions of machine learning models, but it does have some limitations. SHAP values are an approximation of the Shapley values, from which they are based, in order to calculate them efficiently. Even so, SHAP can be computationally expensive and slow for large datasets and complex models. SHAP can only help to explain the fitted model, and so it can only be as good as the model (with caveats around training data containing bias, or incomplete information). Another limitation is the interpretation of SHAP values and its components (the main effect and interactions) can be

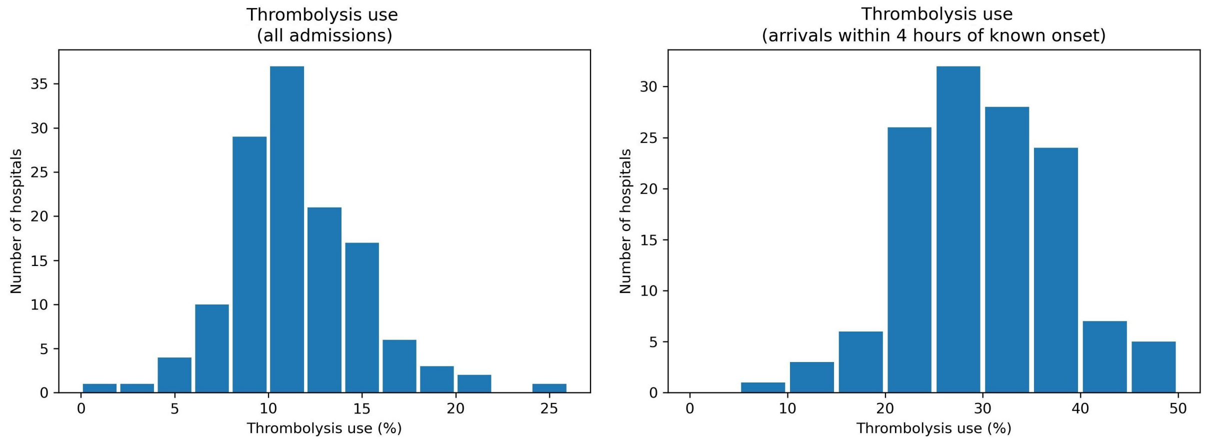

Figure A.1: Histogram of observed thrombolysis use in 132 hospitals. Left: Thrombolysis shown as a percentage of all emergency stroke admissions. Right: Thrombolysis shown as a percentage of those patients who arrive at hospitals within 4 hours of known stroke onset.

challenging. To aid our dissemination of the findings from SHAP we have engaged with clinicians, patients and carers to learn how best to communicate this information. SHAP assumes feature independence - that is the assumption that the values of each feature in a dataset are independent of the values of other features in the same dataset. In other words, the value of one feature should not have a direct effect on the value of another feature. We used feature selection to ensure that very little covariance existed between the 10 features that were included in the models.

A.3 Python libraries

All modelling and analysis was performed using Python in Jupyter Notebooks[1], with general analysis and plotting performed using NumPy[2], Pandas[3], Scikit-Learn[4], and Matplotlib[5].

A.4 Variation in thrombolysis use

Thrombolysis use in the original data varied between hospitals (Figure A.1), from 1.5% to 24.3% of all patients, and 7.3% to 49.7% of patients arriving within 4 hours of known stroke onset.

A.5 Machine learning methods

All work was conducted in Python (v3.8). All code is available at:

1. GitHub repository: `https://github.com/samuel-book/samuel_shap_paper_1`[6]
2. Jupyter book: `https://samuel-book.github.io/samuel_shap_paper_1/`

Our machine learning model used XGBoost (*eXtreme Gradient Boosting*, v1.5, `https://pypi.org/project/xgboost/`). We chose XGBoost for its efficiency, and because we working with a system with known non-linear relationships and potential feature interactions.

We used default settings apart from *learning rate* was set at 0.5 (see section A.12).

Machine learning models were explained using SHAP (*SHapley Additive exPlanations*, v0.41 `https://pypi.org/project/shap/`).

A.6 Feature selection

A simplified model was created by using *forward feature selection* where features were added in accordance to how much each one improved the Receiver Operating Characteristic (ROC) Area Under Curve (AUC). ROC AUC was measured using stratified k-fold validation (k=5). A model with all available 84 features had an ROC AUC of 0.922. A model with 10 features had an ROC AUC of 0.919.

The 10 features selected (Figure A.2) were:

- *Arrival-to-scan time*: Time from arrival at hospital to scan (mins)
- *Infarction*: Stroke type (1 = infarction, 0 = haemorrhage)
- *Stroke severity*: Stroke severity (NIHSS) on arrival
- *Precise onset time*: Onset time type (1 = precise, 0 = best estimate)
- *Prior disability level*: Disability level (modified Rankin Scale) before stroke
- *Stroke team*: Stroke team attended
- *Use of AF anticoagulants*: Use of atrial fibrillation anticoagulant (1 = Yes, 0 = No)
- *Onset-to-arrival time*: Time from onset of stroke to arrival at hospital (mins)
- *Onset during sleep*: Did stroke occur in sleep?
- *Age*: Age (as middle of 5 year age bands)

Stroke team was represented by a one-hot feature vector (e.g. if a patient attended hospital 2 of 5 hospitals, this is encoded as

$$0, 1, 0, 0, 0$$

NOTE: All results from this point forward will use the 10 feature model.

A.7 Correlations within the 10 selected features

Correlations between the 10 features were measured using coefficients of determination (r-squared). All r-squared were less than 0.15, and all r-squared were less than 0.05 except 1) age and prior disability level (r-squared 0.146), and 2) onset during sleep and precise onset time (r-squared 0.078). All correlations are shown in table A.4.

Table A.4: Correlations between the 10 features selected for the XGBoost machine learning model.

Variable 1	Variable 2	r-squared
Age	Prior disability level	0.1462
Onset during sleep	Precise onset time	0.0784
Stroke severity	Prior disability level	0.0454
Stroke severity	Infarction	0.0386
Precise onset time	Onset-to-arrival time	0.0344
Stroke severity	Age	0.0268
Age	Use of AF anticoagulants	0.0207
Stroke severity	Onset-to-arrival time	0.0186
Precise onset time	Prior disability level	0.0131
Age	Precise onset time	0.0090
Prior disability level	Use of AF anticoagulants	0.0070

Table A.4: Correlations between the 10 features selected for the XGBoost machine learning model.

Variable 1	Variable 2	r-squared
Onset during sleep	Onset-to-arrival time	0.0043
Onset-to-arrival time	Age	0.0038
Use of AF anticoagulants	Infarction	0.0033
Prior disability level	Onset-to-arrival time	0.0022
Precise onset time	Arrival-to-scan time	0.0021
Use of AF anticoagulants	Stroke severity	0.0019
Arrival-to-scan time	Stroke severity	0.0019
Precise onset time	Use of AF anticoagulants	0.0016
Stroke severity	Onset during sleep	0.0011
Infarction	Onset-to-arrival time	0.0007
Infarction	Onset during sleep	0.0007
Infarction	Precise onset time	0.0006
Onset-to-arrival time	Arrival-to-scan time	0.0004
Arrival-to-scan time	Prior disability level	0.0001
Onset-to-arrival time	Use of AF anticoagulants	0.0001
Stroke severity	Precise onset time	0.0000
Arrival-to-scan time	Age	0.0000
Use of AF anticoagulants	Onset during sleep	0.0000
Prior disability level	Onset during sleep	0.0000
Infarction	Age	0.0000
Use of AF anticoagulants	Arrival-to-scan time	0.0000
Onset during sleep	Arrival-to-scan time	0.0000
Arrival-to-scan time	Infarction	0.0000
Age	Onset during sleep	0.0000
Prior disability level	Infarction	0.0000

A.8 Model accuracy

Model accuracy was measured using stratified 5-fold cross validation. The key results are shown in table A.5.

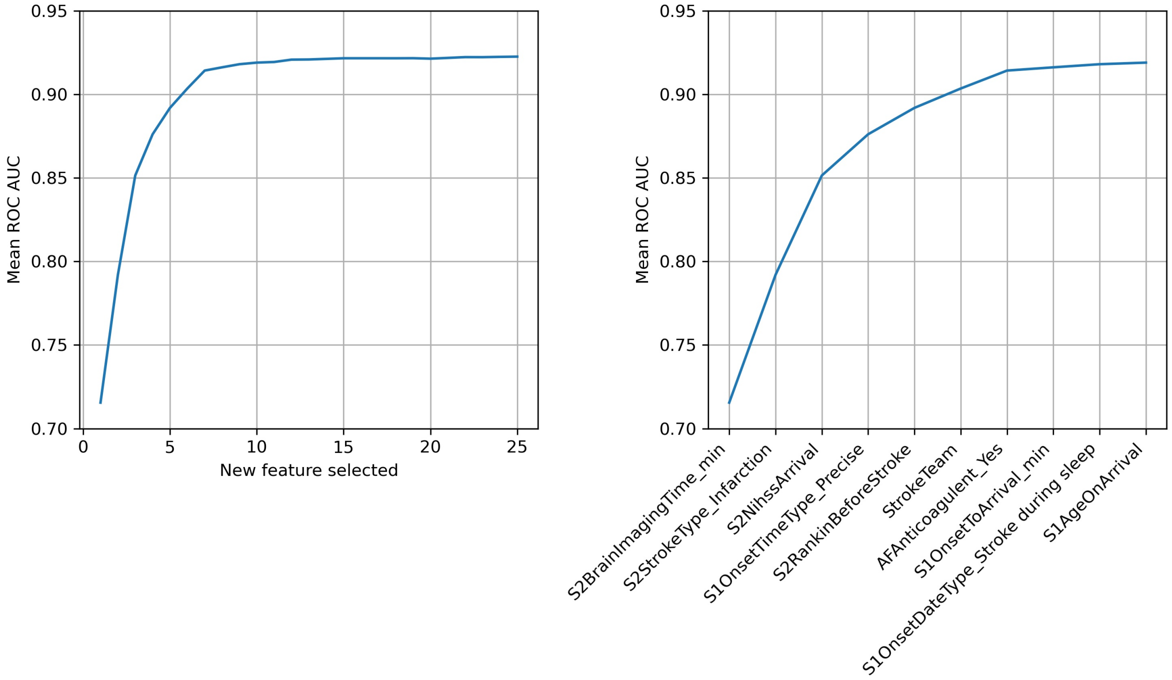

Figure A.2: The effect of increasing the number of features on model accuracy measured by Receiver Operating Characteristic (ROC) Area Under Curve (AUC). Left: Improvement with ROC AUC with selection of up to 25 features. Right: Improvement with ROC AUC with selection of the best 10 features. ROC was measured with stratified 5-fold cross-validation. Results show the mean of the 5-fold replicates.

Table A.5: Accuracy of 10 feature XGBoost model in predicting thrombolysis use in patients arriving at hospital within 4 hours of known stroke onset.

Accuracy measurement	mean	std
Actual positive rate	0.296	0.000
Actual negative rate	0.704	0.000
Predicted positive rate	0.294	0.002
Predicted negative rate	0.706	0.002
Accuracy	0.850	0.004
Sensitivity (recall)	0.743	0.004
Specificity	0.894	0.004
Precision	0.747	0.007
ROC AUC	0.918	0.003
Balanced sensitivity/specificity	0.839	0.003

We found an overall accuracy of 85.0%, with a balanced accuracy. The predicted thrombolysis rate of 29.4% was very close to the observed thrombolysis rate of 29.6%.

Figure A.3 shows the receiver operating characteristic curve, along with the trade-off between sensitivity and specificity.

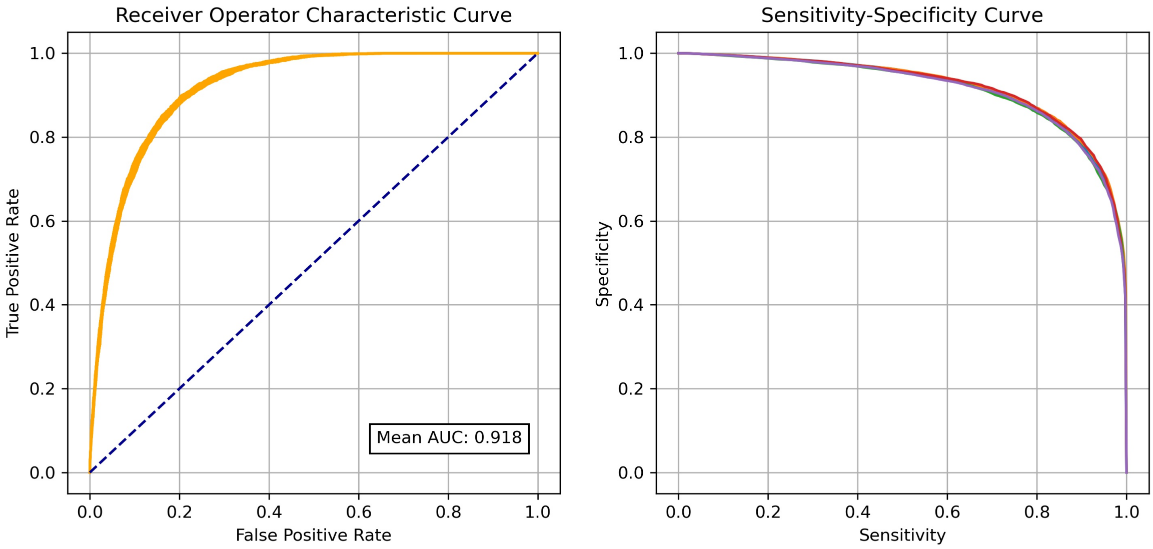

Figure A.3: Model accuracy of a XGBoost model using 10 features. Left: Receiver Operating Characteristic (ROC) Area Under Curve (AUC). Right: The trade-off between Sensitivity and Specificity. Accuracy was measured with stratified 5-fold cross-validation, and both charts show all 5 k-fold replicates.

A.9 Model calibration

The model calibration was checked by binning predictions by probability, and comparing the mean predicted probability with the fraction that were actually positive (table A.6 and figure A.4). In a well-calibrated model, in each bin the average probability of receiving thrombolysis should be close to the proportion of patients who actually received thrombolysis. Results demonstrated that the model was naturally well-calibrated, and was not in need of any calibration correction. As expected, the fraction of predictions that were correct is related to the predicted probability of receiving thrombolysis (when predictions were close to 50% probability of receiving thrombolysis the model was correct about 50% of the time, whereas when the model had predictions of less than 10% or greater than 90% probability of receiving thrombolysis, the model was be correct about 90% of the time).

Nearly 50% of patients fell in the 0-10% probability of receiving thrombolysis - that is the model gave a confident prediction that the these patients would not receive thrombolysis, with the model being correct in these predictions 98% of the time.

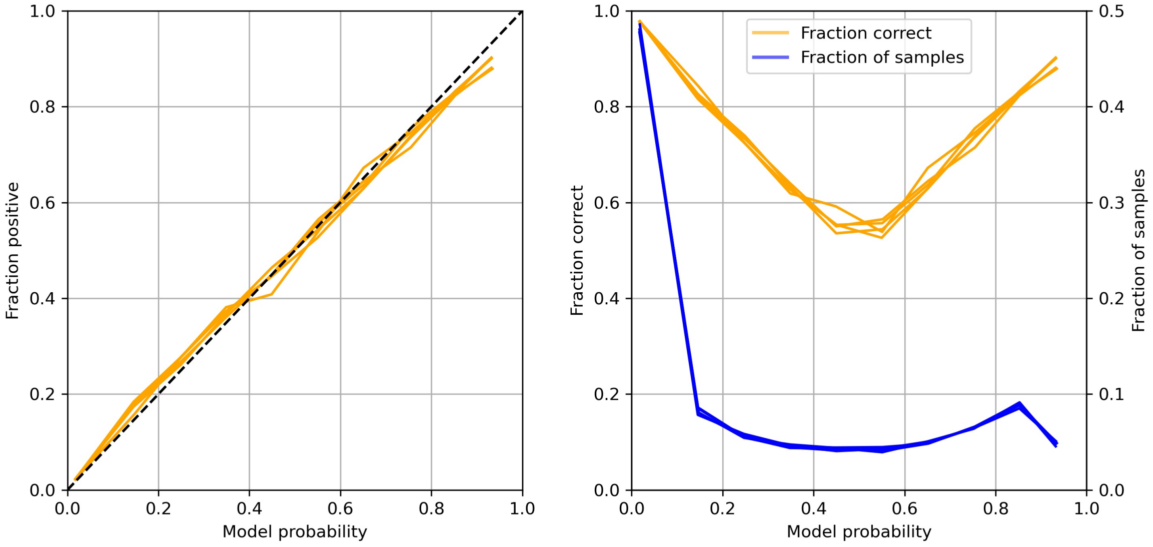

Figure A.4: Calibration check of the model. Left: The proportion of patients receiving thrombolysis for binned probability of receiving thrombolysis. Right: The proportion of predictions in each bin (blue), and the proportion of predictions that are correct (orange). Plot show results for all 5 k-fold replicates.

Table A.6: Model calibration based on binning by predicted probability of thrombolysis.

Bin	Predicted probability	Fraction positive	Fraction correct	Frequency
0.0 - 0.1	0.018	0.023	0.977	0.480
0.1 - 0.2	0.146	0.174	0.826	0.082
0.2 - 0.3	0.248	0.271	0.729	0.056
0.3 - 0.4	0.348	0.371	0.629	0.045
0.4 - 0.5	0.450	0.443	0.557	0.043
0.5 - 0.6	0.551	0.546	0.546	0.042
0.6 - 0.7	0.652	0.643	0.643	0.049
0.7 - 0.8	0.753	0.736	0.736	0.065
0.8 - 0.9	0.852	0.827	0.827	0.089
0.9 - 1.0	0.932	0.893	0.893	0.049

A.10 Evaluating variation in model predictions and predicted 10k cohort thrombolysis rate using bootstrap models

Data was split into a training set of 78,928 patients, and a test set of 10k patients. 30 models were trained, each with a different bootstrap sample of the training set and with a different model random seed. For each of the 10k test set, we evaluated the variation in the predicted probability of receiving thrombolysis (figure A.5). The mean of these standard deviations was 0.057, but the variation depended on the probability, with variation peaking at about 0.13 when the prediction probability of receiving thrombolysis was around 0.5.

Additionally, we used the models and test set to predict thrombolysis use at each of the 132 hospitals if the 10k cohort of patients had attended each of the hospitals (by changing the hospital one-hot encoding, figure A.6). We predicted the thrombolysis use at each hospital, and examined the variation between the

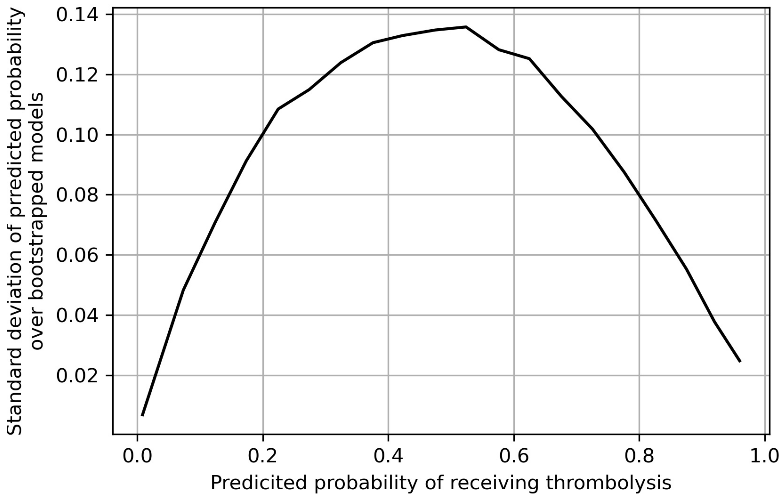

Figure A.5: Standard deviation of predicted probability of receiving thrombolysis, from 30 bootstrapped models predicting the probability of receiving thrombolysis in 10k patients. Results are binned by predicted probability.

30 bootstrapped models. The mean of the standard deviation of bootstrap replicates was 1.7% (where hospital thrombolysis use rates were 10% to 45%).

Bagging experiments were repeated with *Baysian Bootstrapping* based on weighting training samples using a Dirichlet distribution. Very similar results were achieved, with a mean standard deviation of bootstrap replicate probability predictions of 0.054, and a mean standard deviation of bootstrap replicate 10k thrombolysis use in hospitals of 1.6%.

The evaluation of bootstrapped replicates gave us confidence that a single model fit would be sufficient.

A.11 Learning curves

Learning curves evaluate the relationship between training set size and model accuracy. Learning curves were performed using stratified 5-fold validation, and by random sampling (without replacement) of the training set (figure A.7. The maximum accuracy achieved was 85% using 70k training instances, 82.5% accuracy was achieved with 4k training instances. There was a shallow improvement between 4k and 70k training points.

A.12 Fine-tuning of model regularisation

As hospital ID is encoded as one-hot, and there are 132 hospitals, it is possible that the effect of hospitals ID becomes 'regularised out', especially as for each one-hot encoded column about 99

As we are concerned with differences between hospitals, we did not want to over-regularise the model. To optimise *learning rate* we looked at the between-hospital variation of predicted thrombolysis use in a 10k cohort of patients (with the model predicting the use of thrombolysis in each hospital with the same 10k cohort). The model was trained on the remaining 78,928 patients, with varying learning rates (figure A.8 and table A.7).

Reducing the learning rate below 0.5 led to reduced between-hospital variation in the predicted use of thrombolysis, suggesting that the effect of hospital ID was being reduced by over-regularisation.

A learning rate of 0.5 was chosen for all modelling (including the accuracy measurements above).

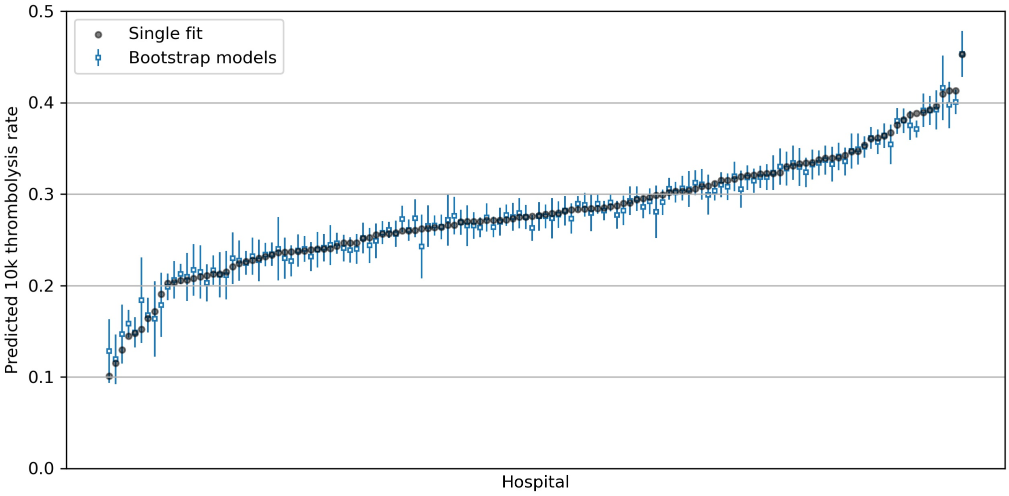

Figure A.6: Mean and standard deviation of predicted thrombolysis use at 132 hospitals from 30 bootstrapped models. Results are for predicted thrombolysis use for the same 10k patient cohort for each hospital. In addition to the results for the bagging models, the predicted thrombolysis use for a single model with bootstrap sampling is shown. Results are ordered by thrombolysis use at each hospital predicted from the single non-bootstrap model.

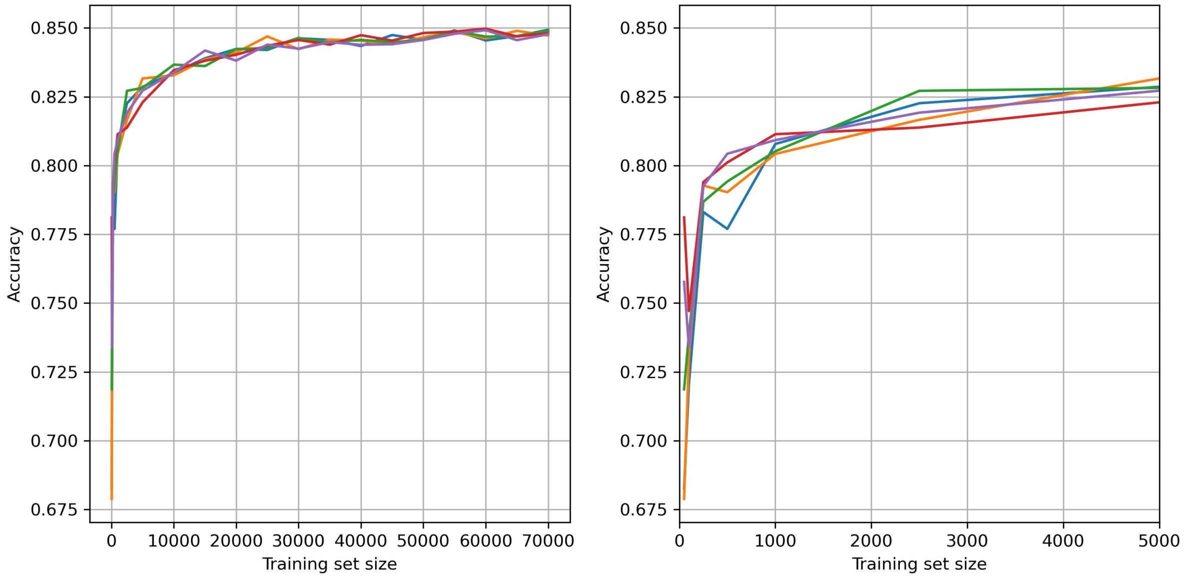

Figure A.7: Learning curves showing the relationship between training set size and model accuracy. Left: training set size up to 70k. Right: training set size up to 5k (same results as the results on the left). Results are shown for all 5 k-fold replicates.

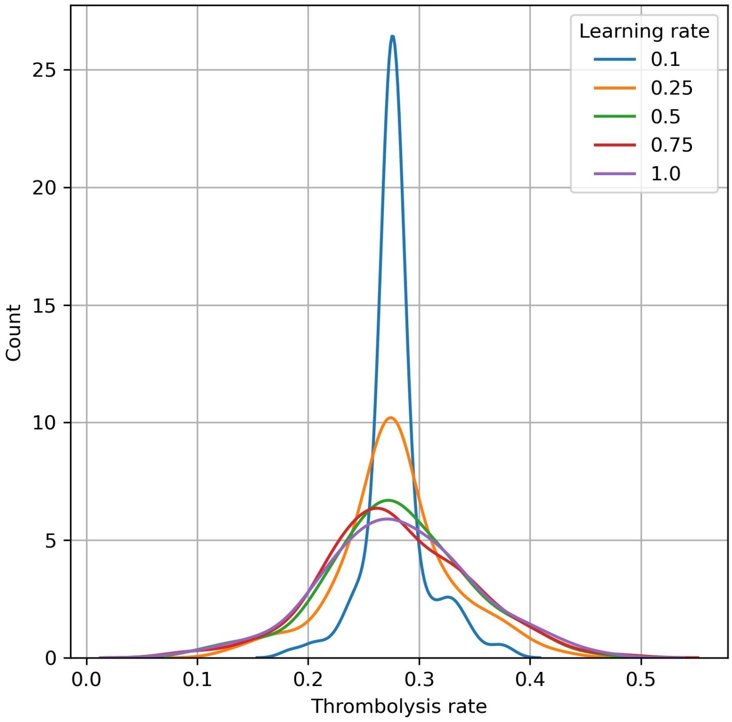

Figure A.8: Effect of adjusting XGBoost learning rate on the distribution predicted thrombolysis use across 132 hospitals. A narrower distribution indicates that hospital thrombolysis rates are tending towards the mean thrombolysis hospital rate.

Table A.7: Statistics on the variation in predicted thrombolysis use between hospitals, with varying learning rate

Learning rate	0.1	0.25	0.5	0.75	1.0
Mean	0.28	0.28	0.28	0.28	0.28
StdDev	0.03	0.05	0.06	0.07	0.07
Min	0.18	0.13	0.10	0.09	0.09
Max	0.38	0.43	0.45	0.48	0.46

Acknowledgements

We would like to thank the SAMueL project team (Lauren Asare, Julia Frost, Iain Lang, Kristin Liabo, Peter McMeekin, Keira Pratt-Boyden, Cathy Pope, Ken Stein, Penny Thompson) for their input into this work.

We would also like to thank our Patient and Carer Involvement team led by Leon Farmer (David Burgess, Simon Douglas, Ian Hancock, Nicola Hancock, John Williams), and our expert advisory group (Ajay Bhalla, Gary Ford, Anthony Rudd, and Martin Utley).

References

[1] Kluyver, T. *et al.* Jupyter Notebooks – a publishing format for reproducible computational workflows. 87–90 (IOS Press, 2016).

[2] Harris, C. R. *et al.* Array programming with NumPy. *Nature* **585**, 357–362 (2020). Number: 7825 Publisher: Nature Publishing Group.

[3] Wes McKinney. Data Structures for Statistical Computing in Python. In Stéfan van der Walt & Jarrod Millman (eds.) *Proceedings of the 9th Python in Science Conference*, 56 – 61 (2010).

[4] Pedregosa, F. *et al.* Scikit-learn: Machine Learning in Python. *Journal of Machine Learning Research* **12**, 2825–2830 (2011).

[5] Hunter, J. D. Matplotlib: A 2D graphics environment. *Computing in Science & Engineering* **9**, 90–95 (2007). Publisher: IEEE COMPUTER SOC.

[6] Pearn, Kerry and Allen, Michael and Laws, and Everson, Richard and James, Martin. samuel-book/samuel_shap_paper_1. `https://github.com/samuel-book/samuel_shap_paper_1` (2023).
